# Chronic Blockade of Brain Endothelin Receptor Type-A (ET_A_) Reduces Blood Pressure and Prevents Catecholaminergic Overactivity in the Right Olfactory Bulb of DOCA-Salt Hypertensive Rats

**DOI:** 10.3390/ijms19030660

**Published:** 2018-02-27

**Authors:** Luis R. Cassinotti, María J. Guil, Mercedes I. Schöller, Mónica P. Navarro, Liliana G. Bianciotti, Marcelo S. Vatta

**Affiliations:** 1 Cátedra de Fisiología, Facultad de Farmacia y Bioquímica, Universidad de Buenos Aires, 1113 Buenos Aires, Argentina; lcassinotti@ffyb.uba.ar (L.R.C.); jguil@ffyb.uba.ar (M.J.G.); mscholler@ffyb.uba.ar (M.I.S.); 2 Instituto de Química y Metabolismo del Fármaco (IQUIMEFA), CONICET—Universidad de Buenos Aires, 1113 Buenos Aires, Argentina; mnavarro@ffyb.uba.ar; 3 Cátedra de Fisiopatología, Facultad de Farmacia y Bioquímica, Universidad de Buenos Aires, 1113 Buenos Aires, Argentina; lbianc@ffyb.uba.ar; 4 Instituto de Inmunología, Genética y Metabolismo (INIGEM), CONICET—Universidad de Buenos Aires, 1113 Buenos Aires, Argentina

**Keywords:** hypertension, systolic blood pressure, endothelin, BQ610, tyrosine hydroxylase

## Abstract

Overactivity of the sympathetic nervous system and central endothelins (ETs) are involved in the development of hypertension. Besides the well-known brain structures involved in the regulation of blood pressure like the hypothalamus or locus coeruleus, evidence suggests that the olfactory bulb (OB) also modulates cardiovascular function. In the present study, we evaluated the interaction between the endothelinergic and catecholaminergic systems in the OB of deoxycorticosterone acetate (DOCA)-salt hypertensive rats. Following brain ET receptor type A (ET_A_) blockade by BQ610 (selective antagonist), transcriptional, traductional, and post-traductional changes in tyrosine hydroxylase (TH) were assessed in the OB of normotensive and DOCA-salt hypertensive rats. Time course variations in systolic blood pressure and heart rate were also registered. Results showed that ET_A_ blockade dose dependently reduced blood pressure in hypertensive rats, but it did not change heart rate. It also prevented the increase in TH activity and expression (mRNA and protein) in the right OB of hypertensive animals. However, ET_A_ blockade did not affect hemodynamics or TH in normotensive animals. Present results support that brain ET_A_ are not involved in blood pressure regulation in normal rats, but they significantly contribute to chronic blood pressure elevation in hypertensive animals. Changes in TH activity and expression were observed in the right but not in the left OB, supporting functional asymmetry, in line with previous studies regarding cardiovascular regulation. Present findings provide further evidence on the role of ETs in the regulation of catecholaminergic activity and the contribution of the right OB to DOCA-salt hypertension.

## 1. Introduction

Hypertension is a multifactorial disease characterized by chronic elevation of systolic and/or diastolic blood pressure (BP) above 140 mmHg and/or 90 mmHg, respectively [1,2]. The etiology and pathophysiological mechanisms involved in the development and maintenance of hypertension remain to be fully elucidated, although strong evidences support a major role for the sympathetic nervous system and the renin angiotensin system. It is well established that neural and non-neural factors contribute to the development of hypertension [3,4]. Neural factors are characterized by an increase in central sympathetic activity. In this sense, ablation of the area postrema or anteroventral third ventricle (AV3V) Brody’s area prevents BP elevation and decreases norepinephrine levels in the brainstem of DOCA-salt rats [5,6]. Non-neural factors include vasoactive peptides such as vasopressin, angiotensin II, natriuretic peptide, and endothelins (ETs) acting at the peripheral or brain level [3,7]. Among the different animal models for hypertension research, the DOCA-salt model is suitable for addressing two of the major pathways involved in hypertension as well as the significant contribution of factors like ETs [8].

ETs comprise a family of three structurally related 21-amino acid peptides (ET-1, ET-2, and ET-3) which are widely distributed in the body, including the central nervous system (CNS) [9,10]. ETs are potent vasoconstrictor substances intimately involved in the regulation of BP and cardiovascular function. In the brain, ETs modulate catecholaminergic transmission, although little is known about their participation in the central regulation of cardiovascular physiology and pathophysiology [9,10,11,12,13,14,15]. ETs mediate their biological effects through two well characterized G-protein coupled receptors, ET_A_ and ET_B_ [10,16,17]. Several authors support the existence of atypical or non-conventional receptors based on the observation of atypical responses to the ET agonists and antagonists that characterize the conventional receptors [13,14,16,18]. However, at present no additional ET receptors have been cloned. Peripheral ET_A_ blockade revealed that this receptor subtype is involved in the origin of chronic BP elevation in different animal models of hypertension [9,10,19]. In contrast, ET_B_ activation has a dual role on the regulation of the vascular tone; it induces vasodilatation through the release of nitric oxide and prostacyclin followed by sustained vasoconstriction [9,16]. ETs applied to the brain increase BP, modify neurohormone release, and augment plasma catecholamine levels [9,10]. However, the role of brain ET receptors in the regulation of BP in both normotensive and hypertensive animals has not been fully elucidated.

The CNS has a key role in the short and long-term regulation of BP [20,21]. ETs and their receptors are expressed in numerous brain regions and areas related to the control of BP and cardiac function like the rostral ventrolateral medulla, nucleus of the solitary tract, the hypothalamus, the locus coeruleus, and the amygdala [9,22]. However, increasing evidence suggests that the olfactory bulb (OB), which expresses ET receptors, also contributes to BP regulation. The OB is an extension of the rostral telencephalon, which projects to the hypothalamus, locus coeruleus, and other brain regions intimately involved in cardiovascular regulation [23,24].

The OB in humans was long considered to be just involved in olfaction, but different reports show that this brain area directly or indirectly contributes to the regulation of functions others than olfaction. Although the OB in humans is smaller than in rats, the number of neurons is similar, and, further, it is even larger in absolute terms [25]. Several studies show that the OB ablation results in critical changes in both dopamine and norepinephrine levels and in the activity of monoamine oxidase in the brainstem and, further, it attenuates cardiovascular sympathoexcitatory reflexes in rats [23,26,27]. A recent work shows that patients with congenital heart disease show subtle dysplasia of discrete brain regions, including the OB, which is associated with delayed brain maturation [28].

We previously reported that ETs applied to the brain modulate noradrenergic transmission (biosynthesis, neuronal release, and uptake of norepinephrine) in the anterior and posterior hypothalamus as well as in the OB of both normotensive and DOCA-salt hypertensive rats [11,12,13,14,15]. In recent studies, we showed that the acute administration (1 h) of an ET_A_ antagonist (BQ610) or an ET_B_ agonist (IRL1620) in the rat brain lateral ventricle modulates tyrosine hydroxylase (TH) expression and its phosphorylated forms in the anterior and posterior hypothalamus as well in the OB. Furthermore, these treatments induce an acute decrease in systolic BP in DOCA-salt hypertensive rats (unpublished observations).

The expression of the endothelinergic system and the presence of catecholaminergic neurons in the OB as well as the extensive network of afferent/efferent connections between this telencephalic structure and areas involved in cardiovascular regulation suggest that the modulation of catecholaminergic activity by ETs in the OB may contribute to BP modulation.

In the present study, we sought to establish the role of ET_A_ in the regulation of catecholaminergic activity in the right and left OB of normotensive and DOCA-salt hypertensive rats. Time course systolic BP and heart rate (HR) were also registered. Catecholaminergic activity in the OB was assessed by possible transcriptional, traductional, and post-traductional changes of TH, which is the rate-limiting enzyme in catecholamine biosynthesis. Studies were performed in the right and left OB to evaluate possible functional asymmetry, as reported by previous cardiovascular studies. Results showed that ET_A_ blockade had no effect on normotensive animals, but it significantly reduced systolic BP and prevented the increase in TH activity and expression in the right OB of hypertensive animals.

## 2. Results

### 2.1. Central ET_A_ Blockade Decreased Systolic BP in DOCA-Salt Hypertensive Rats

**Figure 1:**
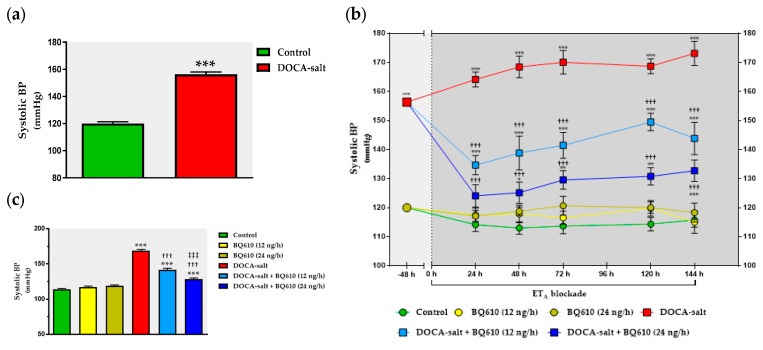
Systolic blood pressure (BP) following ET receptor type A (ET_A_) blockade by BQ610 in normotensive and deoxycorticosterone acetate (DOCA)-salt hypertensive rats. Systolic BP was measured by tail-cuff plethysmography as described in Materials and Methods and expressed in mmHg ± SEM. (**a**) Basal systolic BP. Basal values were registered 48 h before BQ610 administration. *** *p* < 0.001 vs. control. (**b**) Time course of systolic BP. Systolic BP was registered following BQ610 administration at 24, 48, 72, 96, 120, and 144 h. * *p* < 0.05, ** *p* < 0.01, and *** *p* < 0.001 vs. the respective time control; ^†††^ *p* < 0.001 vs. the respective time DOCA-salt. (**c**) Average systolic BP. Each bar represents the area under each curve (shown in **b**) between 0 and 144 h. *** *p* < 0.001 vs. control; ^†††^ *p* < 0.001 vs. DOCA-salt; ^‡‡‡^ *p* < 0.001 vs. DOCA-salt + BQ610. Number of animals: control, 17; BQ610 (12 ng/h), 12; BQ610 (24 ng/h), 8; DOCA-salt, 17; DOCA-salt + BQ610 (12 ng/h), 11; and DOCA-salt + BQ610 (24 ng/h), 10.

**Figure 2:**
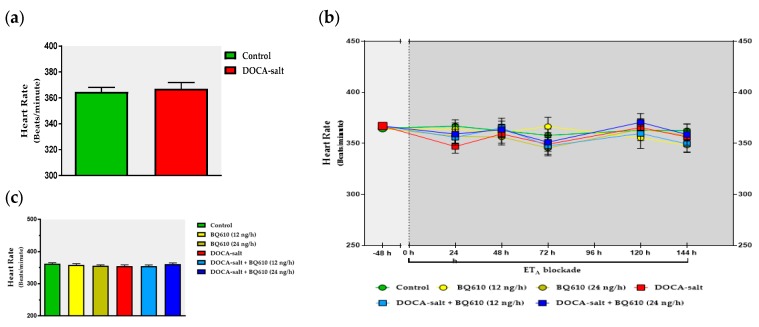
Heart rate (HR) following ET_A_ blockade by BQ610 in both normotensive and DOCA-salt hypertensive rats. HR was assessed as detailed in Materials and Methods and expressed as beats per minute ± SEM. (**a**) Basal HR. Basal values were registered 48 h before BQ610 administration; (**b**) Time course of HR. HR was registered following BQ610 administration at 24, 48, 72, 96, 120, and 144 h; (**c**) Average HR. Each bar represents the area under each curve (shown in **b**) between 0 and 144 h. Number of animals: control, 17; BQ610 (12 ng/h), 12; BQ610 (24 ng/h), 8; DOCA-salt, 17; DOCA-salt + BQ610 (12 ng/h), 11; and DOCA-salt + BQ610 (24 ng/h), 10.

As previously reported, systolic BP was significantly increased in DOCA-salt rats as compared to normotensive animals (156 ± 2 mmHg vs. 120 ± 1 mmHg) (Figure 1). However, no changes in HR (365 ± 3 beats/min (BPM) and DOCA-salt rats 367 ± 5 BPM) were observed in either group (Figure 2a). Blockade of ET_A_ by BQ610 for a week induced no significant modifications in systolic BP or heart rate in normotensive animals (Figure 1b and Figure 2b). In the absence of BQ610, DOCA-salt hypertensive animals further increased systolic BP by 8.3% (169 ± 2 mmHg). Conversely, systolic BP dose-dependently decreased in the presence of the antagonist. BQ610 at 12 ng/h decreased systolic BP by approximately 27 mmHg whereas at 24 ng/h it diminished it by 40 mmHg (Figure 1b). No significant differences were observed in HR following ET_A_ blockade (Figure 2b). Systolic BP and HR values are detailed in Table 1 and Table 2.

### 2.2. ET_A_ Blockade Prevented the Increase in TH Activity in the Right OB of Hypertensive Rats

**Figure 3:**
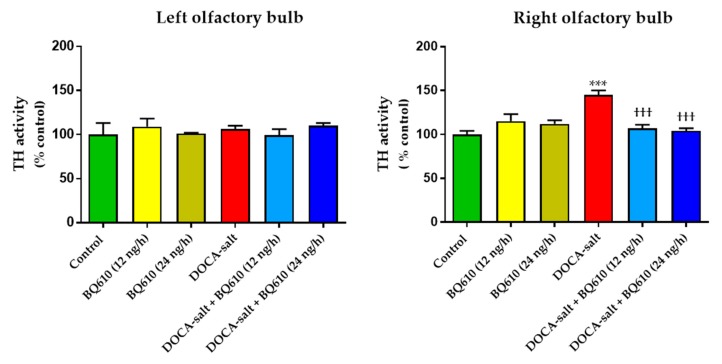
Tyrosine hydroxylase (TH) activity in the left and right olfactory bulb. TH activity was assessed as detailed in Materials and Methods in the left and right OB of normotensive rats (control) and DOCA-salt hypertensive rats. TH activity was expressed as percentage of control ± SEM (% control). *** *p* < 0.001 vs. control; ^†††^ *p* < 0.001 vs. DOCA. Number of animals: control, 5; BQ610 (12 ng/h), 3; BQ610 (24 ng/h), 3; DOCA-salt, 4; DOCA-salt + BQ610 (12 ng/h), 3; and DOCA-salt + BQ610 (24 ng/h), 3.

In order to determine whether ET_A_ blockade affected the catecholaminergic system in the OB, TH activity was assessed (Figure 3). ET_A_ blockade by BQ610 at 12 ng/h or at 24 ng/h induced no changes in TH activity in normotensive animals. DOCA-salt hypertensive rats showed enhanced TH activity in the right OB (~45%), but the increase in the enzyme activity was prevented by ET_A_ blockade (Figure 3). Nevertheless, no changes in TH activity were observed in the left OB of hypertensive animals in the presence or absence of BQ610 (Figure 3).

### 2.3. ET_A_ Blockade Prevented the Increased Expression of TH and Its Phosphorylated Forms in the Right OB of Hypertensive Rats

**Figure 4:**
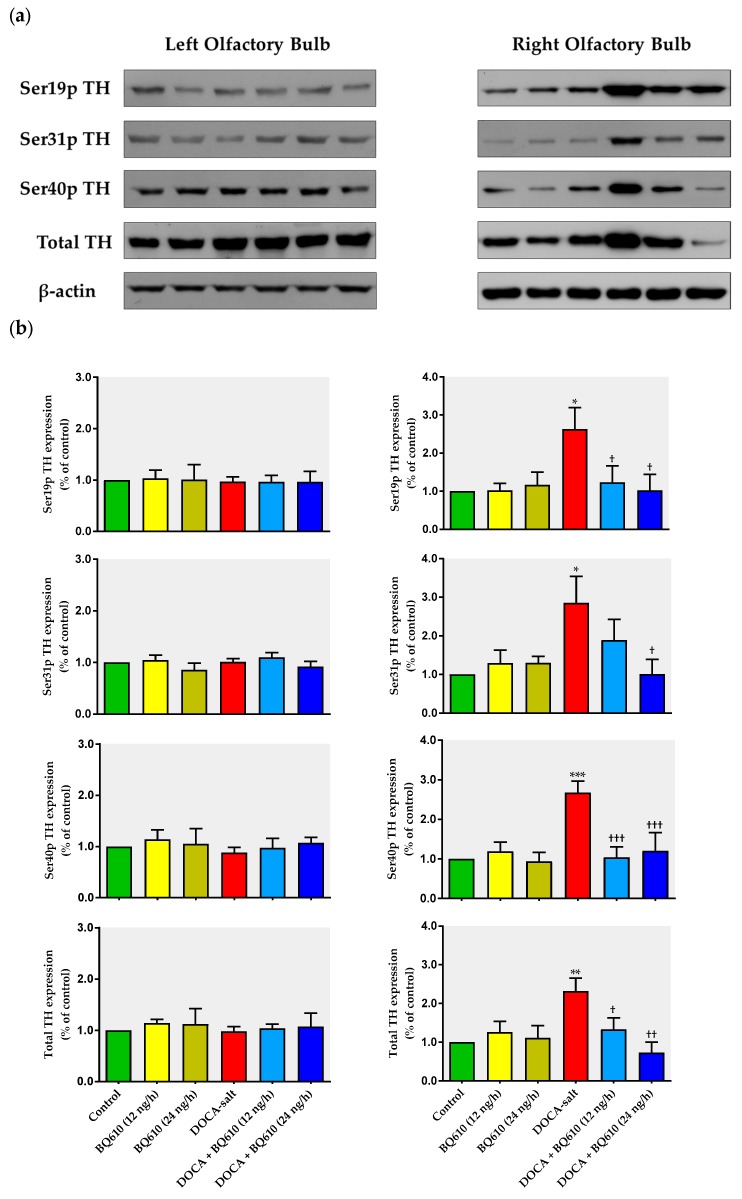
Expression of total tyrosine hydroxylase (TH) and its phosphorylated forms following ET_A_ blockade in the left and right olfactory bulb of normotensive and DOCA-salt hypertensive rats. (**a**) Representative immunoblots; (**b**) Densitometric analysis of TH expression and its phosphorylated levels (Ser19p TH, Ser31p TH, Ser40p TH) in the left and right OB. Results are expressed as percentage of control ± SEM (% control). * *p* < 0.05, ** *p* < 0.01, and *** *p* < 0.001 vs. control; ^†^ *p* < 0.05, ^††^ *p* < 0.01, and ^†††^ *p* < 0.001 vs. DOCA-salt. Number of animals for each experimental group: 4.

We next evaluated whether ET_A_ blockade modified the expression of total TH and phosphorylation at serine 19 (Ser19p), 31 (Ser31p), and 40 (Ser40p) sites. Representative immunoblots are shown in Figure 4a. Blockade of ET_A_ by BQ610 (12 and 24 ng/h) did not modify total TH expression or its phosphorylated forms in the OB of normotensive rats, but it prevented the increase observed in the right OB of DOCA-salt hypertensive animals (Figure 4). However, in the left OB of hypertensive rats, total TH or its phosphorylated forms did not change in the absence or presence of BQ610 (Figure 4b).

### 2.4. Increased TH mRNA Expression in the Right OB of Hypertensive Rats Was Inhibited by ET_A_ Blockade

**Figure 5:**
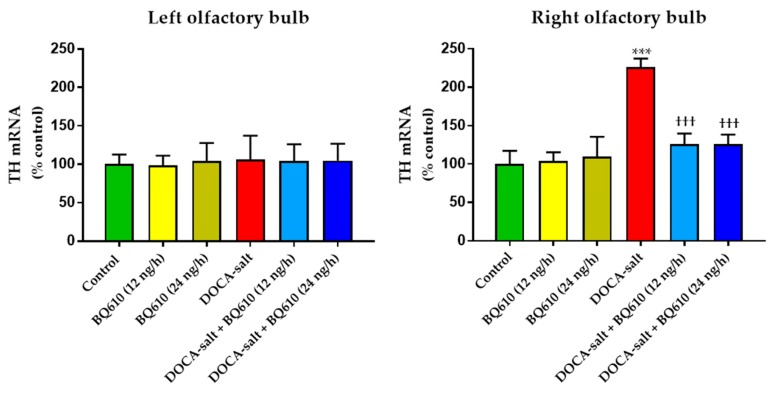
Tyrosine hydroxylase mRNA expression in the left and right olfactory bulb of normotensive and DOCA-salt hypertensive rats. TH mRNA was assessed by real time PCR as detailed in Materials and Methods. Results are expressed as percentage of control ± SEM (% control). *** *p* < 0.001 vs. control; ^†††^ *p* < 0.001 vs. DOCA-salt. Number of animals: 4 in all experimental groups.

TH mRNA expression was assessed in the OB of normotensive and hypertensive rats to evaluate whether long-term ET_A_ blockade affected the catecholaminergic system (Figure 5). ET_A_ inhibition did not affect TH mRNA expression in the OB of normotensive rats. The increase in TH mRNA observed in the OB of DOCA-salt hypertensive rats was prevented by BQ610 at 12 and 24 ng/h (Figure 5). No changes in TH expression were observed in the left OB of hypertensive animals with or without BQ610 (Figure 5).

## 3. Discussion

The present study shows that the brain endothelinergic system is a key factor in the long-term regulation of catecholaminergic transmission in the OB of DOCA-salt hypertensive rats by increasing TH mRNA and protein expression as well TH activity. The major evidence to support it was that central ET_A_ blockade prevented the increase in catecholaminergic activity in the OB of hypertensive rats and, furthermore, it correlated well with the decrease in systolic BP without changes in the HR. These observations together with previous findings support the role of the OB in the progression and maintenance of chronic BP elevation in this animal model of hypertension.

Increasing evidence arising from human and animal studies supports that the overactivation of the sympathetic nervous system and the imbalance of the renin angiotensin system are crucial events involved in the pathogenesis of hypertension. The DOCA-salt model, independent of genetic components, is suitable for studying these two major pathways [8]. In addition, in this animal model ETs play a relevant role in the genesis of chronic BP elevation [3,8,29].

Four different aspects should be considered in the analysis of the present findings: (1) the increase in catecholaminergic activity limited to the right OB; (2) the asymmetrical behavior displayed by OB hemi portions; (3) the effect of brain ET_A_ blockade on the catecholaminergic system in the OB; (4) the effect of ET_A_ blockade on cardiovascular parameters.

The olfactory system, including the OB, is responsible for processing the olfactory information from the external environment. Extensive afferent and efferent connections exist between the olfactory system and other brain areas involved in the regulation of autonomic and behavioral responses, supporting a wide spectrum of biological functions. Increasing evidence supports that the OB contributes in a direct or indirect way to the regulation of cardiovascular function [23,26,27]. The OB integrity is crucial for the CNS to trigger normal sympathoexcitatory responses to diverse physiological stimuli [27]. Moreover, it was shown that cervical stimulation activates α-adrenoreceptors in the OB, leading to venous constriction [30]. Structurally, the OB is formed by several concentric layers. In the glomerular layer, where the integration of sensory information occurs, a great number of gamma-aminobutyric acid (GABA)ergic and dopaminergic neurons coexist, [31]. In this layer, catecholaminergic activity is mediated by dopamine but also by norepinephrine released by nerve endings originated in the locus coeruleus. Enhanced norepinephrine turn-over and changes in the spontaneous firing rate of these neurons are observed in hypertension [26,32,33,34]. The specific marker of catecholaminergic neurons is TH, which catalyzes the rate limiting step in catecholamine biosynthesis [35,36]. Both TH mRNA and protein were reported in the periglomerular neurons of the rat OB [37,38].

Our laboratory was the first to report that ETs enhance noradrenergic transmission in the OB of normotensive rats, supported by short-term increased TH activity and norepinephrine release [39]. In that study, we show that ETs increase TH activity through ET receptor activation coupled to the stimulation of protein kinase A (PKA) and C (PKC), as well as Ca^2+^/calmodulin-dependent protein kinase II (CaMKII). Kinase activation by ETs induces TH phosphorylation at Ser-19 and Ser-40 sites, leading to enhanced TH activity [39]. Moreover, the increase in norepinephrine release stimulated by ETs in the OB suggests that it contributes to enhance TH activity by reducing the feedback inhibition evoked by the amine. In a later study, we reported that long-term exposure to ETs enhances TH mRNA and the expression of total TH and its phosphorylation forms in normotensive rats [14]. Furthermore, to our knowledge, we were the first to report that in the right OB of DOCA-salt hypertensive rats TH activity and expression as well as neuronal norepinephrine release were increased whereas the uptake of the amine was diminished [40].

In the present work, we show that catecholaminergic transmission was enhanced in DOCA-salt hypertensive rats even in the long-term. TH activity was augmented, as supported by enhanced enzyme expression and phosphorylation. The major short-term mechanism of TH activation is protein kinase-induced phosphorylation of serine residues [35,36], whereas the mRNA expression and stability of the enzyme are the main long-term regulatory mechanisms. We show that phosphorylation of TH at Ser-40, 31, and 19 sites was increased in hypertensive animals. Phosphorylation at Ser-40 site substantially increases TH activity, leading to the inhibition of the catecholamine-mediated inhibitory feedback mechanism [35,36]. Ser-40 is considered the most promiscuous site, since it can be phosphorylated by various kinases like PKA, PKC, CaMK-II, and PKG. Phosphorylation at Ser-31 by ERK1 and ERK2 results in a modest increase in TH activity whereas phosphorylation at Ser-19 site by ERK1/2 and CaMK-II results in a Ser-40 enhanced phosphorylation rate due to changes in the enzyme conformation [35,36]. In addition, it was suggested that TH phosphorylation in response to physiological stimuli induces a more active, although less stable, form of the enzyme [35,36]. In the present study, the observation that TH mRNA and total protein expression were enhanced in the right OB of DOCA-salt rats supports positive long-term regulation of the catecholaminergic transmission.

Changes in TH activity were reported in other regions and areas of the CNS in various animal models of hypertension including the DOCA-salt model [15,41,42]. A few reports in the literature address the steps of noradrenergic transmission in the brain of DOCA-salt rats studied herein. In this sense, Rylett et al. [43] reported that TH activity is enhanced in the whole brain, whereas Nagaoka and Lovenberg [44] showed that it is reduced in the hypothalamus. Recent studies from our laboratory show an increase in ET_A_ density, as well as in TH expression in the OB of DOCA-salt hypertensive animals. Furthermore, acute brain ET_A_ blockade (1 h) by BQ610 significantly decreases the expression, phosphorylation, and therefore the activity of TH in the OB of these animals. Also, a differential modulation of catecholaminergic activity in the hypothalamus of DOCA-salt rats occurs due to both short- (1 h) and long-term (7 days) ET_A_ blockade (unpublished observations). These results support present findings. The observed long-term upregulation in catecholaminergic transmission in the right OB may contribute, at least in part, to the development, maintenance, and/or progression of hypertension in this salt-dependent animal model. Furthermore, these results support an excitatory role of this telencephalic region by increasing sympathetic activity.

Present findings also reveal an asymmetrical response between the right and left hemi portions of the OB, with the right hemi-portion being the one that showed important changes, both by DOCA-salt hypertension and chronic ET_A_ treatment (this point will be discussed later). In the left OB, the catecholaminergic activity was unchanged. We previously reported an asymmetrical behavior in the OB of DOCA-salt rats and non-asymmetrical differences in normotensive animals [40]. The existence of brain asymmetry, including norepinephrine levels in the hypothalamus, has been reported in distinct animal species [45]. A study shows the existence of an asymmetrical descending cardiovascular pathway in the dorsomedial hypothalamus supported by the observation that activation of the right OB induces higher HR than activation of the left hemi-portion [46]. An anatomical asymmetry between both hemi-portions of the OB was also shown, where the right side is larger than the left [47]. In regard to hypertension, the present findings are in line with previous studies that support that the right brain region is more associated with the control of cardiovascular function [46,48]. It was shown that patients with essential hypertension evoke higher norepinephrine release in the right jugular vein [49]. Moreover, higher tachycardia is observed upon stimulation of the right sinoatrial node as compared with the left node [50,51]. These studies support that the right sympathetic nervous system would control HR. In addition, the right insular cortex evokes an asymmetrical sympathetic response as compared with the left hemi-portion [48,52]. This report gives further evidence that HR and BP are controlled by the right hemi-portion, since this brain area contains neurons involved in the baroreflex regulation [45,48]. It has also been reported that lipopolysaccharide injection to mice induces sympathoexcitation induced by norepinephrine release in the right region [53]. The right kidney of hypertensive patients without renal stenosis evokes a higher sympathetic discharge than the left [54]. In summary, most studies provide strong evidence that in hypertension, norepinephrine release is increased in right brain areas, thus supporting that cardiovascular regulation is asymmetrical. Although brain functional asymmetry regarding cardiovascular regulation has been widely reported and our results agree in the same way, its physiological relevance remains elusive.

ETs and their receptors are expressed in diverse regions and areas of the CNS including the OB [9,10]. The existence of both endothelinergic and catecholaminergic systems in the OB suggests the modulation of noradrenergic transmission by ETs in this region, as has been shown in other brain areas [11,12,13,14,39]. In the present study, we addressed the impact of chronic (7 days) central ET_A_ blockade on normotensive and hypertensive rats. Results support that the catecholaminergic activity in normotensive animals is not influenced by ETs acting through ET_A_ receptors. However, in hypertensive animals, overactivity of the catecholaminergic activity in the right OB, supported by enhanced TH expression and activity, was prevented by ET_A_ blockade. Our findings strongly support an interaction along time between the endothelinergic and catecholaminergic system in the OB of DOCA-salt hypertensive rats. Catecholaminergic activity seems to be dependent on the central endothelinergic system in hypertension. However, current evidence does not allow us to state whether this relationship is direct or if other mechanisms are also involved.

In the present study, the specific ET_A_ antagonist (BQ610) was applied into the brain ventricle to act on areas where ET receptors are expressed. The OB presents ET_A_ receptors, and the antagonist would reach the OB because the ventricular system of the brain contacts this area. In addition, the cerebrospinal fluid in the rat is close to 300 μL and circulates at an average flow of 132 μL/h, and the volume in the lateral ventricle (approximately 50 μL) is replaced almost three times in that time lapse [55]. Therefore, the antagonist can diffuse assisted by the ventricular microcirculation and the flow caused by the osmotic infusion pump that releases it at a constant rate (0.12 μL/h). However, we cannot assure whether ET_A_ blockade occurs directly in the OB and/or in other brain areas or if our findings result from a more general effect which leads to the modulation of the catecholaminergic activity in this telencephalic region. In any case, we can confirm that brain ET_A_ blockade modulates, even in the long term, the activity of the catecholaminergic system in the OB of DOCA-salt hypertensive animals either in a direct or an indirect way.

To analyze the interaction between the central endothelinergic system and the catecholaminergic transmission in the OB in the context of DOCA-salt hypertension, various aspects should be considered. The OB makes contact with different telencephalic, mesencephalic, diencephalic, and brainstem regions [26], some intimately involved in the control of the cardiovascular function. For instance, the OB interacts with the nucleus of the solitary tract, which is the primary site for the integration of baroreflex input [56,57]. In addition, areas like the amygdala, the septum, the piriform, and orbitofrontal cortex, as well as the ventromedial and posterior nucleus of hypothalamus, connect with the OB. All these areas play an important role in the regulation of cardiovascular function [23,58,59], thus supporting that the OB is likely a relevant actor in this complex mechanism. In this sense, Nagai et al. support the existence of a relationship between olfaction, autonomic balance, and cardiovascular physiology [60]. The exposure of conscious rats to smoke generates changes in BP, breathing, and sympathetic activity [61]. It is important to consider that the OB integrity is necessary in rats to induce normal sympathoexcitatory responses to distinct physiological stimuli. In this sense, bulbectomized rats exhibit an attenuated response to hypotension, endocrine alterations, as well as elevated basal BP and HR [23,24,26,27]. Some authors postulate that the impaired cardiovascular physiology observed under this situation results from changes in the autonomic regulation [62,63]. In this regard, Kelly et al. [26] showed that the cardiovascular changes in bulbectomized rats are associated with changes in the neurochemistry of several neurotransmitters, including norepinephrine. Bilateral olfactory bulbectomy is also a validated animal model of depression that shows diverse functional changes in norepinephrine, dopamine, serotonin, acetylcholine, glutamate, GABA, and monoamine oxidase activity [24,26,29]. It was suggested that this model exerts a strong influence not only on emotions but also on the central control of BP and HR [23]. Although some studies show a bidirectional relationship between depression and cardiovascular diseases, the underlying mechanisms still remain unknown [62,64]. Various studies support that depression leads to the development of cardiovascular diseases by inducing changes in the autonomic regulation [65,66]. In this sense, it was reported that patients with depression exhibit increased HR and sympathetic nervous system activity, as well as baroreflex impairment [64,67].

Several vasoactive peptides, including angiotensin II, natriuretic peptides, and ETs are present in the OB [68,69,70]. ETs behave as neurotransmitters and/or neuromodulators within the CNS. Different works support that the effects of ETs in the brain are mediated by changes in the sympathetic nervous system [9,16]. ETs applied to the brain modify BP, HR, and behavior [9,16]. Although growing evidence supports the participation of the OB in the regulation of BP and cardiovascular activity, little is known about its contribution to hypertension. However, direct evidence suggests that enhanced sympathetic activity is a key factor in DOCA-salt hypertension. In this animal model, plasma catecholamines are increased [3]. In addition, in early and established DOCA-salt hypertension, ganglionic blockade evokes a higher decrease in BP [71]. Peripheral sympathectomy in this animal model prevents enhanced plasma norepinephrine and hypertension [46].

In the present work, the changes in the catecholaminergic activity in the right OB of DOCA-salt hypertensive animals were in accordance with the modifications in systolic BP, further supporting the role of the OB in cardiovascular regulation. Cardiovascular parameters were not affected by ET_A_ blockade in normotensive animals, suggesting that brain ET_A_ receptors are not involved in the physiological control of BP. However, this receptor subtype in the brain appears to have a key role in DOCA-salt hypertension, given that ET_A_ inhibition by BQ610 dose-dependently decreased systolic BP. Although following the administration of the ET_A_ antagonist systolic BP decreased, no changes were observed in HR. It has been previously reported the baroreceptor reflex is impaired in DOCA-salt hypertension [8,72]. It is likely that the fall in BP following ET_A_ antagonist applied to the brain may result from decreased sympathetic activity controlled by the brain, which may in turn affect stroke volume but mainly peripheral vascular resistance. In addition, activation of ET_B_ by endogenous ETs could play a role in this mechanism. However, the underlying mechanism(s) that induce(s) BP decrease by brain ET_A_ blockade remains to be established.

Taken together, in consideration of previous and present findings, it is possible to hypothesize the existence of a close and strong relationship between the central endothelinergic system and the asymmetric catecholaminergic transmission in the OB of hypertensives rats. This interaction would play an important role in the brain modulation of the cardiovascular function in DOCA-salt hypertension. The present study presents evidence for the role of the OB in salt-dependent hypertension. However, additional experimental and clinical studies are necessary to prove that the present findings may have clinical implications in humans. Understanding the role of brain areas indirectly or directly related to the regulation of cardiovascular function that have been relegated or poorly studied may favor not only the scientific knowledge itself but also the development of new therapeutic tools for the prevention and/or treatment of hypertension, a disease which nowadays exhibits greatest morbidity and mortality on a global scale.

## 4. Materials and Methods

### 4.1. Animals and Experimental Design

Seventy-five Sprague-Dawley male rats with an approximate body weight of 150 g were used (Animal Facility of the School of Pharmacy and Biochemistry, University of Buenos Aires, Buenos Aires, Argentina). Animals were housed in steel cages and maintained under constant temperature and humidity conditions, with 12 h light/dark cycles and food provided ad libitum under the supervision of a professional Technician. All experiments were performed following the principles of the Guide for Care and Use of Laboratory Animals and the International Guiding for Biomedical Research Involving Animals [73,74]. All experiments were approved by the Institutional Committee on Care and Use of Laboratory Animals (CICUAL) at the School of Pharmacy and Biochemistry, University of Buenos Aires (Note 031013-5; Exp-FYB N° 56106/2013, 8 August 2013). All efforts were made to minimize the number of rats and stress caused.

Animals were randomly divided into two groups: control (normotensive) and DOCA-salt. DOCA-salt hypertension was induced by two subcutaneous weekly injections of 15 mg/kg of deoxycorticosterone acetate (DOCA) (MP Biomedicals, Santa Ana, CA, USA) dissolved in sesame seed oil (vehicle) and by administration of 1% NaCl in water for five weeks. Normotensive control animals were injected with vehicle and drank tap water. Body weight and water consumption were determined twice a week for all rats. Also, systolic BP and HR were registered by Non-Invasive Blood Pressure (NIBP) controller and PowerLab 8/30 data acquisition (ADInstruments Model 125 and Model ML 870, respectively; Pty Ltd., Sydney, Australia) using a Pulse Transducer for Rat (Panlab/Harvard Apparatus, Barcelona, Spain). One week before the administration of the first DOCA injection, animals were manipulated by the operator who eventually administered the DOCA and made BP recording.

BP measurements were performed in a quiet room where animals were previously acclimatized for a 1-h period. To minimize stress, no restraint tubes were used, but a dark cover was placed over the animals [75,76]. Daily, the operator manipulated the animal, placed it on a heating pad (32–34 °C), put a dark blanket over the animal, placed the occlusion cuff on the animal, and, when the animal was quiet, inflated the cuff. In our experience, with this habituation protocol, no restraint tubes are necessary because the animal gets familiar with the operator and the procedure. As supported by the literature, methodology considerations optimize the operative capacity to measure systolic BP and HR in normal or experimental conditions by tail-cuff plethysmography [75,77]. As advised, three to eight measurements per animal were registered [75]. Measurements were recorded between 10 a.m. to 2 p.m. to avoid circadian variations [78]. It is important to consider that if the methodology recommendations for the tail cuff method are followed, the values of systolic BP and HR are similar to those obtained by telemetry [79,80].

At week 4, animals were anesthetized with ketamine/xylazine (80/10 mg/kg, IP) and underwent intracerebroventricular (ICV) stereotaxic surgery (David Kopf Mod. 900, Tujunga, CA, USA). ICV surgery was performed according to The Rat Brain at Stereotaxic Coordinates [81] and using a brain infusion kit (B.I.K.1, DURECT Co., Cupertino, CA, USA). The cannula was kept connected all time to an osmotic microinfusion pump (ALZET model 1004; DURECT Co.) located subcutaneously in the middle scapular region of the animal. The osmotic microinfusion pump vehiculized the ET_A_ antagonist BQ610 at two different concentrations: 100 ng/μL (constant flow 12 ng/h) or 200 ng/μL (constant flow 24 ng/h) for 7 days continuously, in both cases dissolved in artificial cerebrospinal fluid (aCSF, vehicle) of a composition recommended by ALZET, DURECT Co. One-third of the animals in each group received osmotic microinfusion pumps loaded with 100 μL of BQ610 (12 ng/h), another third loaded with 100 μL of BQ610 (24 ng/h), and the remaining third with a pump loaded with 100 μL of aCSF. In this way, six different treatments were formed (Table 3).

At 1 h following surgery and for 48 h, animals orally received proper analgesia and antibiotic therapy. Systolic and HR were recorded at 24, 48, 72, 120, and 144 h. At week 5, animals were euthanized by decapitation, brains were removed, and right and left OB were dissected according to Maps and guide to microdissection of the rat brain [82]. Samples were processed for total RNA and proteins extraction, or for TH activity determination.

### 4.2. Methods

#### 4.2.1. TH Activity 

Tissues were homogenized in 200 μL lysis buffer (5 mM KH_2_PO_4_ and 2% Triton 100X, pH 7.0). Cell debris was removed by centrifugation at 12,500 rpm for 10 min at 4 °C. An aliquot of the supernatant was saved for protein quantification. TH activity was determined by the technique described by Reinhard et al. [83]. Briefly, samples were incubated for 20 min at 37 °C with the reaction solution (50 mM HEPES, pH 7.0; 15 nmol ^3^H-l-tyrosine (PerkinElmer Life and Analytical Sciences, Billerica, MA, USA), 420 mM β-mercaptoethanol, and 1000 U catalase). The reaction was stopped by cold and an aliquot of the reaction mixture was placed in 1 N HCl with an activated charcoal suspension (7.5% *w*/*v*). Following centrifugation for 10 min at 4 °C, supernatants were recovered to measure ^3^H_2_O by liquid scintigraphy (WinSpectral 1414 counter, Perkin Elmer/Wallac, Ramsey, MN, USA). Results were expressed as percentage of control ± SEM.

#### 4.2.2. Tissue Homogenization for Western Blot Assay and RT-PCR

Total RNA and proteins from OB were isolated by an extraction kit according to the manufacturer’s specifications (TRIreagent, Molecular Research Center Inc., Cincinnati, OH, USA). To improve the protein yield isolated by this method, an additional resuspension step was made by using a urea 9.5 M (Biopack, Buenos Aires, Argentina) in 2% buffer CHAPS solution (Thermo Fischer Scientific, Waltham, MA, USA).

#### 4.2.3. Western Blot Assay for TH

Experimental procedures for Western blot were as previously detailed [84]. Samples separated on SDS-PAGE and electro-transferred to PVDF membranes (GE Healthcare, Amersham Biosciences, Little Chalfont, UK) were then exposed to primary antibodies overnight at 4 °C, followed by horseradish peroxidase (HRP)-conjugated anti-rabbit or anti-mouse antibodies for 1 h at room temperature. Membranes were developed by a bioluminescent Western blotting detection system (Kalium Technologies, Buenos Aires, Argentina) and exposed to X-ray films. Bands were analyzed by densitometry and normalized to β-actin. Primary and peroxidase conjugated secondary antibodies and dilutions are shown in Table 4.

#### 4.2.4. Real-Time PCR for mRNA TH

Experimental procedures for mRNA TH were as previously detailed [13] with minor modifications. Residual DNA co-purified with total RNA was removed by incubation with DNase (TransGen Biotech, Beijing, China) for 60 min at 37 °C. Total RNA quality and quantity was assessed by 1% (*w*/*v*) agarose gel electrophoresis and UV spectrometry, respectively. An amount of 2 μg RNA was retrotranscribed to cDNA with reverse transcriptase MMLV (Genbiotech, Buenos Aires, Argentina) and RNase inhibitor (RNasin, Genbiotech, Argentina), dNTPs, and oligo-dT primer (IDT, San Jose, CA, USA). TH or β-actin (housekeeping) mRNA expression were analyzed in a RotorGene Q-series thermocycler (QUIAGEN, Hilden, Germany). The oligonucleotide sequences used as primers are summarized in Table 5.

TH cDNA amplification product corresponds to an 81 bp fragment between nucleotides 1184–1264 in the original mRNA sequence, whereas the β-actin is 69 bp and is between nucleotides 1090 and 1158 of the original mRNA sequence. Reactions were performed with cDNA, Evagreen fluorochrome (Biotium, San Francisco, CA, USA), TH, or β-actin specific primer mix (IDT, San Jose, CA, USA), 0.6 μL MgCl_2_ 50 mM, 0.4 μL dNTPs 10 mM, 4 μL 5× GoTaq buffer, and 0.3 μL GoTaq DNA polymerase (Promega, Madison, WI, USA) in a 20 μL final volume. Amplification consisted of 35–40 cycles (denaturation at 94 °C for 15 s, hybridization at 54 °C for 45 s, and extension at 72 °C for 30 s). For each sample, sequential monitoring once per cycle at the end of extension was performed. Samples were measured in duplicate. For each primer pair, an external standard RNA concentration curve was done using pooled RNA samples and confirmed by agarose gel electrophoresis. Melting curve analysis showed the presence of a single cDNA product per primer pair, thus confirming the specificity of PCR products. Specificity was further confirmed by agarose gel electrophoresis that showed a single band of the predicted size for each product. Minor variability among samples were corrected by normalization of TH expression to β-actin. Relative expression quantification was carried out by the standard curve method. Results were expressed as percentage of control ± SEM. Sequences of primer pairs are summarized in Table 5.

### 4.3. Statistical Analysis

The statistical analysis was performed using *t*-test, 1W-ANOVA followed by the Newman-Keuls comparisons, or 2W-ANOVA followed by the Bonferroni’s comparisons according to the requirement of each experiment. *p*-values of 0.05 or less were considered statistically significant.

## Figures and Tables

**Table 1:** Systolic blood pressure (BP) values following ET receptor type A (ET_A_) blockade. Systolic BP was expressed as mmHg ± SEM. C, normotensive control; DOCA, DOCA-salt hypertensive rats.

Experimental Groups	Systolic BP Following ET_A_ Blockade by BQ610				
	24 h	48 h	72 h	120 h	144 h
Control	114.1 ± 2.3	113.0 ± 2.2	113.7 ± 2.7	114.4 ± 2.3	115.8 ± 2.5
C + BQ610 (12 ng/h)	117.5 ± 2.4 NS	117.9 ± 3.0 NS	116.6 ± 2.3 NS	119.8 ± 2.7 NS	115.1 ± 3.9 NS
C + BQ610 (24 ng/h)	117.2 ± 1.9 NS	118.8 ± 1.5 NS	120.7 ± 3.2 NS	120.1 ± 1.9 NS	118.3 ± 3.3 NS
DOCA-salt	164.1 ± 2.6 ***	168.5 ± 3.7 ***	170.1 ± 4.1 ***	168.7 ± 2.6 ***	173.1 ± 4.2 ***
DOCA + BQ610 (12 ng/h)	134.7 ± 3.3 ***^,†††^	138.8 ± 5.8 ***^,†††^	141.5 ± 4.5 ***^,†††^	149.5 ± 3.0 ***^,†††^	143.9 ± 5.6 ***^,†††^
DOCA + BQ610 (24 ng/h)	124.1 ± 3.8 ^†††^	125.2 ± 3.7 *^,†††^	129.6 ± 3.2 **^,†††^	130.8 ± 3.0 **^,†††^	132.8 ± 3.7 ***^,†††^

* *p* < 0.05, ** *p* < 0.01, and *** *p* < 0.001 vs. control; ^†††^ *p* < 0.001 vs. DOCA-salt. Number of animals: control, 17; BQ610 (12 ng/h), 12; BQ610 (24 ng/h), 8; DOCA-salt, 17; DOCA-salt + BQ610 (12 ng/h), 11; and DOCA-salt + BQ610 (24 ng/h), 10. NS: Not significant.

**Table 2:** Heart rate (HR) values following ET_A_ blockade. HR is expressed as beats per minute (BPM) ± SEM. C, normotensive control; DOCA, DOCA-salt hypertensive rats. Number of animals: control, 17; BQ610 (12 ng/h), 12; BQ610 (24 ng/h), 8; DOCA-salt, 17; DOCA-salt + BQ610 (12 ng/h), 11; and DOCA-salt + BQ610 (24 ng/h), 10.

Experimental Groups	HR During Long-Term ET_A_ Antagonist Treatment				
	24 h	48 h	72 h	120 h	144 h
Control	367 ± 6	362 ± 6	358 ± 6	363 ± 6	363 ± 6
C + BQ610 (12 ng/h)	364 ± 5	361 ± 5	367 ± 9	355 ± 10	348 ± 7
C + BQ610 (24 ng/h)	357 ± 7	356 ± 8	345 ± 3	364 ± 5	358 ± 5
DOCA-salt	347 ± 7	359 ± 9	349 ± 11	366 ± 9	356 ± 8
DOCA + BQ610 (12 ng/h)	356 ± 6	365 ± 9	348 ± 8	360 ± 7	349 ± 8
DOCA + BQ610 (24 ng/h)	359 ± 11	364 ± 8	351 ± 7	371 ± 9	359 ± 10

**Table 3:** Experimental groups used in the present study.

Groups	ET_A_ Antagonist/Vehicle	Doses
Control	Vehicle	
Control + BQ610	BQ610	12 ng/h
Control + BQ610	BQ610	24 ng/h
DOCA-salt	Vehicle	
DOCA-salt + BQ610	BQ610	12 ng/h
DOCA-salt + BQ610	BQ610	24 ng/h

**Table 4:** Antibodies used for western blot assays. Primary and peroxidase-conjugated secondary antibodies. Catalogue numbers are shown between brackets.

**Primary Antibody**	**Dilution**	**Company**
Total TH monoclonal (mouse)	1:2000	Sigma, St. Louis, MO, USA (T1299)
Ser19p TH polyclonal (rabbit)	1:4000	Chemicon, Millipore Co., Temecula, CA, USA (AB5425)
Ser31p TH polyclonal (rabbit)	1:750	Chemicon, Millipore Co., Temecula, CA, USA (AB5423)
Ser40p TH polyclonal (rabbit)	1:2000	Chemicon, Millipore Co., Temecula, CA, USA (AB5935)
β-actin monoclonal (mouse)	1:1000	Sigma, St. Louis, MO, USA (A2066)
**Secondary Antibody**	**Dilution**	**Company**
Anti-mouse (Goat) (for Total TH)	1:5000	Santa Cruz, Dallas, TX, USA (sc2005)
Anti-rabbit (Goat) (for Ser19p TH)	1:7500	Chemicon, Millipore Co., Temecula, CA, USA (AP307P)
Anti-rabbit (Goat) (for Ser31p TH)	1:1500	Chemicon, Millipore Co., Temecula, CA, USA (AP307P)
Anti-rabbit (Goat) (for Ser40p TH)	1:5000	Chemicon, Millipore Co., Temecula, CA, USA (AP307P)
Anti-mouse (Goat) (for β-actin)	1:4000	Santa Cruz, Dallas, TX, USA (sc2005)

**Table 5:** Primers sequences used for real time PCR.

Primers	Nucleotide Sequences	GeneBank Accession Number
TH forward	5′-AGGGCTGCTGTCTTCCTAC-3′	NM_012740
TH reverse	5′-GCTGTGTCTGGGTCAAAGG-3′	
β-actin forward	5′-TCTGTGTGGATTGGTGGCTCTA-3′	NM_031144.3
β-actin reverse	5′-CTGCTTGCTGATCCACATCTG-3′	

## Abbreviations

aCSF	Artificial cerebrospinal fluid
BP	Blood pressure
CNS	Central nervous system
ETs	Endothelins
ET_A_	Endothelin receptor type A
ET_B_	Endothelin receptor type B
HR	Heart rate
OB	Olfactory bulb
TH	Tyrosine hydroxylase
